# Professor Vacca's Memorial on the Recto-Vesical Method of Extracting Urinary Calculi, with Cases

**Published:** 1821-09-01

**Authors:** 


*Memoria sopra il Metodo, &c. Memorial on the Method of extracting the Stone from the Urinary Bladder by the Rectum.* By ANDREW VACCA BERLINGHIERI, Prof. of Clinical Surgery in the University of Pisa, &c.

WE were the first, we believe, to call the attention of our countrymen to the new method of performing the operation of lithotomy, originally proposed by Mr. Sanson, a French surgeon, in our observations on a case where that method was adopted by Prof. Barbantini of Lucca.* On perusing Prof. B's account of the *recto-vesical* operation, by which our attention was first called to the subject, we were forcibly impressed with the advantages which it appeared to us to possess over both the *lateral* and *high* operations; and in our notice of Prof. B's memorial, we ventured to express our opinion, and to recommend in strong terms the consideration of the subject to English surgeons. What impression our sentiments, on the recital of the case alluded to, made on the minds of our readers, we know not; but we now feel much satisfaction in being able to shew that we were not singular in our opinions, since they seem to coincide with those formed and acted upon by one of the most celebrated Italian surgeons of the present day, Professor Vacca of Pisa, of whose interesting Memorial, published a few months ago, we shall now proceed to give as clear and succinct an analysis as we can.

Professor Vacca is already too well known to the medical world to require any encomiums from us;—his present Memorial, we believe, will not detract from his fame. He

---

* See our Journal for April 1820, No. 8, p. 631.

begins by disclaiming all credit in the invention of the *recto-vesical* operation, and gives the whole merit of the discovery to Mr. Sanson; shewing by a quotation from Vigetius, who was a veterinary surgeon, that he speaks only of extracting urinary calculi, by the rectum, from brutes, when the bladder has been accidentally ruptured: he anticipates, however, a greater degree of success than the original proposer had, in calling the attention of surgeons to the *Sansonian operation*, from his being able to support it by more numerous and satisfactory cases.

"I think," he says, "I can shew by not a few observations what simple reasoning induced Mr. Sanson to believe, that the *recto-vesical* operation for the stone deserves by every title a preference to all others, not indeed as recommended by Mr. Sanson, or as performed by Mr. Dupuytren, and several Italian surgeons, but by a different method, which Mr. Sanson, however, described as possible."

Our author next takes a view of the different methods that have been adopted for extracting calculi from the bladder, and of the advantages and disadvantages attending each; through the whole of this we need not follow him, but shall merely notice his opinion of the only two methods that are likely to be put in competition with that under consideration —the *high* and *lateral* operations. Of the first of these Prof. V. at one time thought very highly and frequently practised it, but more extensive experience has induced him to change his opinion. The great danger of peritoneal inflammation and of the extravasation of the urine, independent of the difficulty which often arises in the performance of this operation from the thickness and contractions of the recti muscles, and contracted state of bladder over large stones, are more than sufficient, our author thinks, to counterbalance the only two advantages which the high operation possesses over all other methods known before 1817, that of enabling us to extract stones of any size, and of certainly avoiding injury of the important blood-vessels. Prof. V's objections to the *lateral* operation are its exposing the patient to the risk of hæmorrhage, or an injury of the rectum; to the danger arising from the size of the stone, requiring, either that it should be broken, or the patient subjected to another operation. The two first occurrences our author allows may by care be avoided, and that the necessity for another operation may be a rare occurrence, but he has often experienced the difficulty himself, and felt for the prolonged suffering of his patients, in breaking down and extracting large stones. "Hence," he concludes, "that although the incision in this operation may be easily and speedily performed, the extraction of the stone may become a laborious, long, and painful process—presenting difficulties which have been felt by all the ablest lithotomists, as the works of those who know alike how to publish their successful and unsuccessful operations sufficiently attest;" p. 25; and we may add, as all who have seen but a few operations for lithotomy performed must be fully impressed with—who have witnessed the sufferings of the unfortunate patient, tortured under the attempts of the surgeon to extract a stone too large to pass, except by bruising and lacerating the parts through which it is dragged, or the tedious and painful grapling and digging to extract its broken fragments. Surely if there is a shocking operation in surgery it is this; and most sincerely do we congratulate surgeons on the prospect of their being exempted from the performance of so painful a task, and mankind in general from such dreadful sufferings.

Of the superiority of the new method we shall let our author speak for himself.

"The recto-vesical operation," he observes, "seems to unite all the principal advantages and to present the fewest inconveniences. I need not call to the minds of my readers their knowledge of minute anatomy to persuade them that there is no point of the perineum nearer to the bladder than that which is immediately anterior to the sphincter ani. There is not much ingenuity required to perceive, that by cutting in this point the sphincter, the parietes of the rectum, the membranous part of the urethra, and the prostate, by an incision, which divides but few soft parts, we shall have procured an opening sufficiently large for the entrance of our fingers, our forceps, and the egress of the stone; because we take advantage of the natural aperture of the anus and of the cavity of the rectum. The most general notions of anatomy suffice to convince us that an incision which divides the anterior part of the sphincter ani, the membranous part of the urethra, in the middle line of its anterior (INFERIOR) parietes, the neck of the bladder, the prostate and fundus of the bladder in the same line, does not approach any large vessel or other part important to life. It is clear also that by this method the stone will pass between the rami of the ischium when they are very far separated, and when they in consequence have an ample space for the passage of the largest stones. It is also evident, that the direction and shortness of the wound render impossible any extravasation of urine; render the wound itself less extensive, and the exit of any fragments of stone that may remain in the bladder more easy.

"The depth of the wound being less than by the other methods, the surgeon is enabled to penetrate farther into the bladder with his finger to ascertain the form, size, and situation of the stone previous to his grasping it for extraction.

"By the *recto-vesical* operation, then, we avoid the danger of wounding the large vessels of the perineum; and, as in the high operation, we are enabled to extract the largest stones, without the inconvenience of laying bare the peritoneum, and of exposing the surgeon to the risk of wounding it, or of the danger of extravasation of urine." P. 25, &c.

The objections that may be stated to the *recto-vesical* operation are chiefly two—its wounding the rectum, and secondly, its establishing a communication between the rectum and bladder, and admitting the contents of one cavity into the other, by which a sterco-urinary fistula may be formed. To the *first* objection in the present state of our knowledge of the structure of the rectum, an answer is scarely required. The *second* appears a much more weighty objection; our author answers it however satisfactorily.

"This inconvenience is not a *necessary* attendant on the extraction of the stone by the rectum, but of the *peculiar method* to which Mr. Sanson seems to give the preference, and which Mr. Dupuytren and other surgeons have adopted. It may easily be avoided by dividing the urethra, the prostate and neck of the bladder *only*. By this process the incision of the intestine is at least an inch lower than that of the neck of the bladder;—the parietes also of the neck of the bladder remain in contact, being separated only during the passage of urine; by this method also the parietes of the intestine perform the office of a valve, which prevents the passage of the fæces into the bladder." P. 32.

In support of this the Professor refers to his cases.

The description of our author's method of operating is rather tedious: we shall give the essentials. The instruments required are a common grooved staff, a common straight bistoury, a pair of forceps, and, in some cases, a very narrow straight bistoury, its point terminating in a small button. The patient is placed and secured in the same way as for the common lateral operation. The staff is first introduced and given to an assistant, who takes care to keep it firm, the handle perpendicular to the pubis, neither inclining it to one side or other, but so that the groove part shall correspond to the middle line of the urethra or raphe. The flat edge of the bistoury being closely applied along the palmar aspect of the index finger of the left hand, to avoid the intestine being wounded during the introduction, they are to be carefully introduced into the rectum, the back of the finger being directed towards the sacrum, its palmar aspect, upon which is applied the bistoury, towards the symphysis pubis. In this way they are to be carried into the intestine to the distance of ten or twelve lines: the finger is then to be pressed backwards towards the sacrum, so as to admit of the cutting edge of the bistoury being turned towards anterior part of rectum, its back now resting on the finger; taking care that the cutting edge of the bistoury points directly to the raphe of the perineum ; the index finger, upon which it rests, is now to press the bistoury forwards, so that it shall divide the anterior part of the intestine; with the right hand the bistoury is then to be withdrawn, so as to continue the incision, then begun in the rectum, through the cellular substance between it and the urethra, the external sphincter of the anus and beyond it, not more than eight or nine lines, in the perineum. The first incision being thus finished, the index finger of the left hand is to be introduced into the wound, immediately beyond the divided sphincter, and the groove of the staff sought for with the nail, (which it is said should always be long when the surgeon performs this operation)—this being felt through the parietes of the urethra, the same bistoury is to be introduced guided by the nail of the finger, so as to divide the urethra, the nail and point of the bistoury being now in the groove of the staff, still retained firmly in its original situation, the bistoury is to be pushed forward along the groove into the bladder, so as to divide the prostate and neck of that viscus to a greater or less extent, according to the operator's idea of the size and form of the stone. As we are very apt however to err on this subject, the Professor thinks it will be better to make rather a small wound of the neck of the bladder and prostate, as, if necessary, it may be easily enlarged by the straight bistoury, having the small button at its extremity. We need not follow our author through the remaining part of the operation, which is the same in this as in the lateral. With regard to the dressing of the wound, Prof. V. enters a strong protest against introducing any substance whatever between the edges; an unnecessary piece of advice this, we believe, for English surgeons. Frequent washing, so as to maintain the parts perfectly clean, is all that is required. To allay the pain, opium given in small or large doses, according to circumstances, diluting mucilaginous drinks, the application of leeches about the anus, and in the robust even copious general bleeding, with the strictest diet, till all danger of inflammation is over, are the means used by the Professor.

The danger of inflammation being over, and suppuration established, our author thinks it necessary to begin to touch that part of the wound which corresponds to the incision of the rectum, and that portion which remains in the perineum, with lunar caustic. This practice is said to accelerate the cicatrization in a wonderful manner, and in the only one of his patients where the wound was not speedily cured, he attributes the circumstance to the neglect of this practice by his assistant, he himself being obliged to leave the hospital for some time. Professor Vacca thinks the *vagino-vesical* operation equally applicable to women as the *recto-vesical* to men; though, in the former, the fundus of the bladder should be cut, and the neck of that viscus and urethra avoided. One case of stone in a woman has alone presented itself to our author, since he became acquainted with the new method; urgent symptoms compelled him to operate, but her pregnancy rendered it, in his opinion, imprudent to do it by the vagina.

We now come to Professor Vacca's cases, which are reported by his pupils, and as we consider them very interesting, we shall give a short notice of each.

The *first* is a patient of 70 years of age, much emaciated. Having endeavoured to allay the irritation which this poor man suffered from the stone and a stricture of the urethra, the operation was performed on the 7th of February, 1820; a purgative of cream of tartar having been administered the day before, (a practice which is followed in the school of Pisa before all great operations,) and the rectum cleared by an injection the morning of the operation. The operation was executed with ease The extraction of the stone, however, presented great difficulties; it was of a large cylindrical form, the extremities pointing to the ischia; from this position it was found impossible to move it; it was therefore broken and extracted in three pieces; the stone was rough, and to one of the fragments adhered some soft membranous filaments. This last circumstance induced the Professor to think that the stone had been adherent to the bladder. Tepid water was injected into the bladder to remove any fragments that might remain. The operation thus finished, the patient was laid in bed, and as he had lost very little blood, and suffered much, eight leeches were immediately applied to the perineum, and four over the pubis.—Twenty drops of laudanum were given—fomentations ordered to be applied to the hypogastrium, and the patient kept on the lowest diet, having abundance of diluent drinks. In the evening the irritation was calmed, but a slight fever had appeared; urine mostly by wound, a small quantity only by natural passage. Next day pain and tension of hypogastrium with increase of fever. Day following symptoms still increased; more leeches applied. Towards evening of this third day after operation he died. On examination, peritoneum was found inflamed, coats of bladder much thickened, and a puriform fluid effused between muscular coat and peritoneal, also into cellular membrane between bladder and pubis. Internal membrane of the bladder appeared gangrenous, and some fragments of the stone were found attached where probably the stone had adhered. The liver much enlarged.

*Second case* was a healthy country boy of five years old—operated on May 4th, 1820—whole operation performed with ease—six leeches applied round anus immediately after—towards evening slight fever with uneasiness of hypogastrium to which four more leeches were applied. On fourth day after operation a few drops of urine passed by urethra, but it was not till the eleventh that it came regularly in small quantity by urethra, and continued to augment; on the 20th June this boy was discharged with a small fistula in perineo, from which a few drops of urine escaped every time he passed his urine. This, as we have already noticed, Prof. V. attributed to the application of the caustic being neglected. The boy after some time was taken back to the clinical wards, and when Prof. V's observations were published, a small fistula still remained.

*Third case* was a country-man, aged 38 years, of a cachectic habit, who had suffered for two years, about which time the calculus seemed to have passed from the pelvis of the kidney into the bladder. This patient had his symptoms much increased by a rough journey of fifty miles; a few days after his arrival in clinical ward, he was attacked by a dysenteric affection. This affection having abated after some time, though there still remained some fever, urine charged with blood and mucus and the alvine evacuations continuing about seven a day, the operation was resolved on, from the urgent entreaties of the patient, and the hope that the dysenteric symptoms, &c. might be symptomatic of the presence of the calculus in the bladder. The incision and extraction of the stone, which was rough and of the size of the largest pigeon's egg, were performed with facility. Twenty drops of laudanum were given. Day after operation fever was less than day before it was performed, the alvine evacuations were fewer, urine almost entirely by wound, a few drops only by penis. Second day free from fever, though towards evening it returned with tension and pain of hypogastrium; eight leeches and fomentations were applied to this region, though patient was very weak—alvine evacuations much diminished; urine, partly from wound, partly by natural passage, less charged with blood and mucus. From this time patient went on well, urine passing entirely by wound till eighth day, when a few drops began again to pass by urethra, and the quantity continuing to augment daily; on the fifteenth the whole urine flowed by the natural passage when patient was in horizontal posture, though a few drops passed by wound in the erect posture. Sixteenth day, slight œdema of face and lower extremities appeared without any evident cause. Eighteenth, urine came entirely by natural passage in erect posture. Twenty-fifth day from operation wound almost cicatrized, though slight degree of œdema still remained. August 30th, discharged cured from hospital, thirty days after operation. From fifth day after operation, whole extent of incision touched with caustic.

*Fourth case* occurred in a healthy countryman, aged 74 years, whose age seemed the only objection to operation. The preparatory ounce of cream of tartar being administered, as usual, on preceding day, this patient was also submitted to operation on 1st August, 1820, after the exhibition of an enema. Nearly fifty calculi, varying from the size of a pea, to that of a small nut, were extracted in this case, and with these a small pediculated cyst containing a limpid fluid. Immediately after operation twenty drops of laudanum were given, and eight leeches applied to perineum; the lowest diet and copious dilution enjoined. Towards evening, fever with some tension of belly appearing, eight ounces of blood were taken from arm, and emollient fomentations applied. Next morning he was free from fever, which however returned with chills about eleven o'clock; and in the evening eight ounces more blood were taken from arm. The fever continuing to recur daily, without any pain or tension of abdomen, was suspected to be of the intermitting kind, and bark was administered, which speedily stopped it. On the eighth, urine began to pass by penis in small quantity, increasing daily by this way till 20th, when it passed entirely by natural passage. On 25th, sphincter, which is always *last part* of wound to heal, had nearly cicatrized, and on 30th he was discharged completely cured.

*Fifth case*, a hatmaker, aged 38, of robust constitution.—Operation performed with ease, Sept. 24th, 1820—stone, from its friability, broke, which rendered extraction rather tedious. Fourteen ounces of blood were taken from arm immediately after operation. October 6th, urine passed entirely by penis—9th, a few drops again passed from wound for last time. In this case also intermittent fever shewed itself, and yielded to bark in a few days.* October 27th, discharged cured.

---

* Both these patients, we believe, must have suffered at some former period from intermitting fever, which in them had been reproduced by

The *sixth* and last case was a child two years old. The operation was performed with ease. A few drops of blood only were lost. Urine from first flowed in part from urethra. On 9th day after operation not a drop passed by wound. October 28th, discharged cured from hospital. Operation was performed October 14th.

Such are the cases operated on by himself, which Prof. Vacca brings forward in support of his opinion of the *recto-vesical* operation, and which cannot fail to have very considerable weight, we imagine, in calling the serious attention of surgeons to the subject. In the perusal of these cases we remarked with pleasure the great attention which Prof. V. seems to pay to his patients after his operations, differing in this respect from some operators who seem to think the operation the only thing deserving of their attention—conduct which cannot be too severely reprobated, and of which, if we mistake not, it has been our misfortune to witness more than once the fatal effects—when after a fairly performed operation, the patient has been lost from inattention to the supervening symptoms.

To his own cases Prof. V. adds a brief account of five others, which have been performed in Italy, one by Dr. Farnese, a surgeon in Milan. Patient, in this case, was a healthy man of 50 years. The urethra, neck of bladder, and prostate, were divided; not the fundus of the bladder. The symptoms were mild—no feculent matter passed off with the urine, which, after 10th or 12th day, passed entirely by natural passage;—the wound was frequently touched by cautic, and in 33 days was quite cured. Dr. F. is about to publish an account of this case, with a memorial on the operation. Two cases have also been operated on with success by Prof. Geri, of Turin; they are published, though our author had not seen them. He knew, however, that in the first operation the fundus of the bladder was divided, and that feculent matter passed off by the urethra, with the urine; notwithstanding which, the patient was

---

the irritation of system from the operation. In countries subject to these fevers it is very common to observe the inflammatory diseases of winter, pneumonia, rheumatism, &c. accompanied by intermittent fever, in those subjects who have laboured under that disease during the preceding autumn, forming in these cases what may be called *symptomatic intermittent fever*, differing, however, from symptomatic fevers in frequently continuing after the disease which excited it has been removed, and requiring the administration of bark to remove it. Whatever excites febrile irritation in such ague-tainted constitutions, seems capable of reproducing this fever, though it does not follow that the removal of this irritation will put an end to this capricious fever.

cured in a short time. The manner of operating adopted by Signor Geri, in his second case, our author does not know. The two remaining operations were performed by Prof. Barbantini, of Lucca; the result of the first we laid before our readers in a former number of our Journal. The fundus of the bladder, it will be recollected, was divided in that case, and the fæces entered that viscus and passed off by urethra; the patient had a small urinary fistula left. Prof. B's second operation was performed in the same way, and was one of those cases where an operation is performed as the only remaining, though almost hopeless, resource. The symptoms for first two days after operation were mild;—the feculent matter found its way into the bladder, and passed off with the urine. Peritoneal inflammation supervened and carried off the patient. On examination after death extensive organic disease of the bladder was observed; and much feculent matter had passed into that viscus. The case is not yet published.

The *recto-vesical* operation then has been performed in Italy, as far as we know, in *eleven* cases—of which *seven* have proved completely successful. In *two*, urinary fistulæ have remained as a sequel of the operation; and in *two* instances the operation has been followed by the death of the patient. It is to be observed, however, that both these were very unpromising cases; and that their death is probably in no way to be attributed to the mode in which the operation was performed. In the one case extensive organic disease of the bladder, which had been strongly suspected before the operation, was found to exist after death; and the other was a debilitated subject seventy years of age, who for twenty-four years had suffered from affections of the urinary organs; and where the extraction of the stone was rendered peculiarly difficult from its position, and its adhesion to the internal coat of the bladder, which itself appears also to have been in a diseased state previous to the operation.

With regard to the peculiar method of performing the Sansonian operation, all our present information appears in favour of that adopted by our author. In both cases the operation seems performed with equal facility, but the latter does not appear to be liable to the very disagreeable occurrence of the fæces finding their way into the bladder, which seems always to be the case in the former.

"It is proved," says Prof. V. "from all the observations now known on the subject, that the passage of the feculent matter from the intestine into the bladder occurs *only* where the fundus of the bladder is divided, the neck of that viscus, the prostate, and urethra remaining untouched, as we have before stated; and *never* where the neck of the bladder has been divided, the fundus being respected, as was done by Sig. Farnese, and as I have constantly done."

A moment's reflection on the relative position and structure of the parts concerned in this operation will sufficiently explain why the fæces are likely always to find their way into the bladder in the one method of operating, and not in the other. Prof. V. however, is not of opinion that the incision should, in every case, be confined to the neck of the bladder and prostate; he conceives it much better, where the stone is found of a very large size, to extend the incision into the fundus of the bladder, than to lacerate the parts by its forcible extraction. In this we perfectly agree with our author: the only risk arising from this extension of the incision for a short distance into the fundus of the bladder, is the contents of the rectum finding their way into the former viscus; but unless the incisions were carried far back, we do not think it near so likely that they would, as when the incision is entirely made in the fundus vesicæ.

We have now brought to a conclusion our analysis of Prof. Vacca's Memorial, of which it has been our object to give as full an account as our limits would admit, from the importance which we attach to the subject. Prof. Vacca has our sincere thanks for the information he has given us, and which we have taken the earliest opportunity of laying before our readers—thus forwarding the object of his Memorial, which was to make more generally known in Italy the new method of operating, with the reasonings and observations upon which it was supported. If that distinguished surgeon has not, in the present instance, the merit of discovering, he certainly has that of improving, an operation, which, without having seen performed, we cannot help expressing our belief will, at no very distant period, supersede all other operations for the extraction of the stone. It seems to us, with our author, to possess all the advantages of the other methods of operating, with fewer inconveniences than any of them. The operation itself seems of easy performance, and void of any danger to the patient, as far as the incisions are concerned. It allows abundant space for the extraction of the largest stone, without bruising or lacerating the wounded parts; (this we consider its greatest advantage;) and hence obviates the necessity, in any case, of breaking down the stone—often a difficult, always a dangerous and painful operation; and when the stone, from its friable nature, is accidentally broken, the direct and depending opening which it presents will greatly facilitate the extraction of the broken fragments, and thus diminish the danger of nuclei for new concretions being left in the bladder.

